# Establishment of reverse transcription recombinase-aided amplification with lateral flow dipstick for the rapid visual detection of Getah virus

**DOI:** 10.3389/fcimb.2025.1631048

**Published:** 2025-08-13

**Authors:** Bingyan Wei, Lulu Xing, Siqi Sun, Pu Zhang, Yanjun Dong, Hanchun Yang

**Affiliations:** ^1^ State Key Laboratory of Veterinary Public Health and Safety, College of Veterinary Medicine, China Agricultural University, Beijing, China; ^2^ Key Laboratory of Animal Epidemiology of Ministry of Agriculture and Rural Affairs, College of Veterinary Medicine, China Agricultural University, Beijing, China

**Keywords:** Getah virus, arbovirus, recombinase-aided amplification, lateral flow dipstick, nucleic acid detection

## Abstract

**Introduction:**

Getah virus (GETV) is a globally spreading zoonotic mosquito-borne virus that primarily affects horses and pigs, causing significant economic losses in the livestock industry. Therefore, there is an urgent need for improved diagnostic methods to manage future outbreaks.

**Methods:**

In this study, we developed a nucleic acid detection assay, reverse transcription recombinase-aided amplification with lateral flow dipstick (RT-RAA-LFD), for the rapid and convenient detection of GETV.

**Results:**

The RT-RAA-LFD assay could be completed at 40°C for 15 min. Under optimal reaction conditions, the assay demonstrated excellent specificity, with no cross-reactivity observed with other clinically relevant swine pathogens. It achieved a broad detection range and a limit of detection (LOD) of 5.53 × 10^2^ copies/μL, which was lower than that of RT-PCR (5.53 × 103 copies/μL) assay and slightly higher than that of qRT-PCR (5.53 × 10^1^ copies/μL) assay for GETV. When testing 21 blood samples, the results of RT-RAA-LFD were fully consistent with those of the RT-PCR and qRT-PCR assays. In testing 45 tissue samples, the Kappa value for consistency between RT-RAA-LFD and RT-PCR was 0.776 (P < 0.001), with a concordance rate of 95.6% (43/45). The Kappa value for consistency between RT-RAA-LFD and qRT-PCR was 0.845 (P < 0.001), with a concordance rate of 97.8% (44/45).

**Conclusions:**

In conclusion, the RT-RAA-LFD assay shows great potential as a efficient and user-friendly diagnostic tool for GETV screening, particularly in laboratories with limited resources and equipment.

## 1 Introduction

Getah virus infection is a mosquito−borne zoonosis caused by Getah virus (GETV) that circulates among vertebrates (Karabatsos, 1978; Hubálek et al., 2014). The virus has a wide range of susceptible hosts, including humans and multiple vertebrate animals such as livestock and poultry (e.g., horses, pigs, cattle, chickens, and ducks), laboratory animals (e.g., rats, guinea pigs, mice, monkeys, rabbits, and orangutans), as well as wildlife (e.g., kangaroos, blue foxes, and red pandas) (Fukunaga et al., 1981; Lu et al., 2020; Shi et al., 2022). Among these, horses and pigs are the primary hosts. Infected horses typically present with fever, rash, limb edema, and lymphadenopathy, while infected pigs exhibit symptoms such as abortion, hind limb paralysis, diarrhea, tremors, and reproductive disorders in sows (Nemoto et al., 2015; Liu et al., 2019; Lu et al., 2019; Zhao et al., 2023). GETV was initially isolated from mosquitoes in Malaysia in 1955 (Morita and Igarashi, 1984). Since then, GETV has gradually spread across Eurasia and the Pan-Pacific (Shi et al., 2022). In China, GETV was first identified and isolated in Hainan Province in 1964 (Li et al., 1992). In June 2017, a GETV outbreak occurred on a pig farm in Hunan Province, resulting in the miscarriage or mummification of over 150 pregnant sows and the death of approximately 200 piglets (Yang et al., 2018). By 2022, GETV had been detected in more than 22 of the 34 provincial-level administrative divisions (Li et al., 2022). An epidemiological investigation conducted from 2022 to 2023 involving bovine sera collected in Yunnan Province, on the China-Myanmar border, revealed positive rates of GETV antibodies and RNA of 20.25% and 0.23%, respectively (Liu et al., 2023). Additionally, a separate epidemiological survey carried out in Jiangxi Province indicated that 95.65% (44/46) of pig farms and 47.93% (197/411) of samples tested positive for GETV (Lan et al., 2024). From late July to mid-September 2024, a highly virulent GETV variant caused concentrated outbreaks among pig farms across multiple regions of Henan Province, leading to significantly increased mortality in piglets (Sun et al., 2025). With the expanding geographical distribution of GETV, significant economic losses have been caused to the livestock industry in China. Therefore, strengthening rapid clinical diagnostics and routine surveillance of GETV is critical for early detection, source control and effective containment of future GETV outbreaks.

Currently, the main detection methods for GETV include viral isolation, serological assays, and molecular diagnostics. Viral isolation remains one of the most accurate laboratory diagnostic methods. However, its clinical utility is limited by the prolonged time required for viral growth, low isolation rates, and the need for high−level biosafety facilities and specialized expertise, making it impractical for routine use in primary settings (Cao et al., 2022). Serological tests such as enzyme-linked immunosorbent assay (ELISA) have several limitations, including long turnaround times, stringent serum quality requirements, and susceptibility to false-positive results (Cao et al., 2022). Molecular diagnostic methods based on reverse transcription polymerase chain reaction (RT-PCR) and real-time RT-PCR amplify cDNA generated from viral RNA. These methods, characterized by their accuracy, efficiency, and high specificity, are currently the principal techniques for pathogen identification (Jian et al., 2024). Nevertheless, they require specialized instrumentation, longer reaction times, and skilled personnel to operate (Lan et al., 2025).

**Figure 1:**
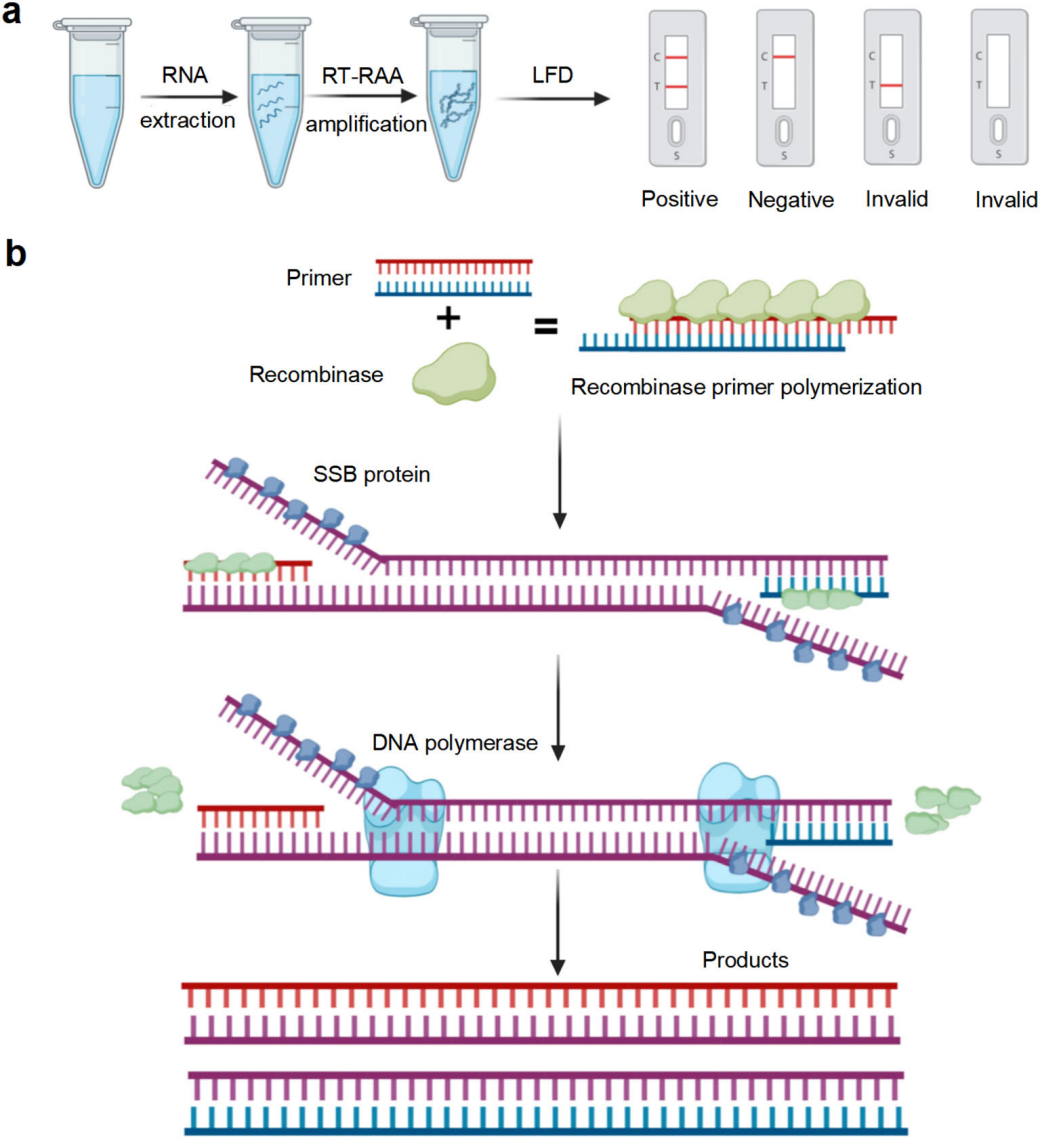
Illustration of reverse transcription recombinase-aided amplification with lateral flow dipstick (RT-RAA-LFD) reaction. **(a)** Flowchart of the RT-RAA-LFD reactions. **(b)** Schematic representation of the RT-RAA reaction. Created in BioRender.

The recombinase-based isothermal amplification technologies provide a rapid, user−friendly and portable alternative with high sensitivity and specificity, making them suitable methods for on-site pathogen surveillance. Recombinase polymerase amplification (RPA), developed by TwistDx (Cambridge, UK), and recombinase-aided amplification (RAA), developed by Qitian (Wuxi, China), are representative diagnostic tools that enable rapid and specific detection of diverse pathogens (Fan et al., 2020). Both rely on three core proteins: recombinase, single-stranded DNA binding protein (SSB), and DNA polymerase. The RPA/RAA reaction depends on three core proteins: recombinase, single-stranded DNA-binding protein (SSB), and DNA polymerase. The difference between RAA and RPA is the source of recombinase. RAA utilizes recombinase obtained from bacteria or fungi, whereas RPA employs recombinase derived from phage T4, which is less readily available (Wu et al., 2022). The recombinase mediates sequence−specific pairing of oligonucleotide primers with the target region, the SSB stabilizes the resulting single-stranded DNA, and the polymerase drives exponential amplification of the selected fragment (Mao et al., 2022). RAA reactions can be completed within 15-30 min under constant temperatures ranging from 37 to 42°C, enabling rapid amplification of DNA or RNA templates (Lin et al., 2022). Amplification products of RAA assays can be detected using agarose gel electrophoresis, lateral flow dipsticks, and real-time fluorescence assays. The principle of the RAA reaction is illustrated in **Figure 1**.

In this study, we aimed to develop a rapid GETV RT-RAA-LFD assay by combining reverse transcription recombinase-aided amplification (RT-RAA) with lateral flow dipstick (LFD) for the diagnosis of GETV infection in resource−limited settings.

## 2 Materials and methods

### 2.1 Viruses and experimentally infected samples

The Getah virus (GETV) strain (GenBank accession No. PP537546), porcine epidemic diarrhea virus (PEDV) strain (GenBank accession No. KX066126.1), porcine deltacoronavirus (PDCoV) strain (GenBank accession No. MG832584.1), inactivated classical swine fever virus (CSFV) strain (GenBank accession No. Z46258.1), senecavirus A (SVA) strain (GenBank accession No. MN433300.1), porcine circovirus type 2 (PCV2) strain (GenBank accession No. AF538325.1), and pseudorabies virus (PRV) strain (GenBank accession No. KU057086.1) were obtained from the Key Laboratory of Animal Epidemiology, Ministry of Agriculture and Rural Affairs. In an experiment conducted in the laboratory, 30 blood samples were collected from ICR mice on days 1, 3, 5, and 7 following inoculation with the GETV NM2022 strain. Additionally, 60 tissue samples were collected on day 7. All animal procedures were approved by the Laboratory Animal Welfare and Animal Experimental Ethical Committee of China Agricultural University, Beijing, China (No. AW922024202-2-1).

### 2.2 Nucleic acid extraction

The blood samples were left to stand at 4°C for 12 h and then centrifuged at 3000 rpm for 10 min at the same temperature. Total RNA was extracted from serum, liver, spleen, kidney, and brain tissue samples using TRIzol reagent (Thermo Fisher Scientific, Waltham, MA, USA). cDNA synthesis was performed with the HiScript III qRT SuperMix kit (Vazyme Biotech Co. Ltd., Nanjing, China) according to the manufacturer’s recommendations. Briefly, 4 µL of 4 × gDNA Wiper Mix and 1 µg of total RNA were combined with RNase-free water to a volume of 16 µL, and the mixture was incubated in a T100 Thermal Cycler (Bio-Rad, Hercules, CA, USA) at 42°C for 2 min to eliminate genomic DNA. Subsequently, 4 µL of 5 × HiScript III qRT SuperMix was added, bringing the final reaction volume to 20 µL. Reverse transcription was carried out at 37°C for 15 min followed by 85°C for 5 s. The resulting cDNA was stored at -20°C.

### 2.3 Preparation of recombinant plasmid

PCR amplification of the GETV Cap gene was performed using the cDNA synthesized by reverse transcription as a template. The purified amplicon was ligated into the pMD19-T vector with the pMD19-T Vector Cloning Kit (Baori Doctor Biotechnology Co. Ltd., Beijing, China) according to the manufacturer’s instructions. A 10 µL ligation mixture, including 1 μL of pMD19-T, 5 μL of Solution I, 3 μL of insert DNA, and 1 μL of ddH_2_O, was incubated at 16°C for 30 min in a T100 thermal cycler. The ligation products were transformed into competent E. coli cells, and transformants were selected on antibiotic agar plates. Subsequently, individual colonies were randomly picked, cultured in liquid medium, and verified by PCR. PCR Positive bacterial cultures were sequenced by Sangon Biotech Co. Ltd. (Shanghai, China). The validated recombinant plasmid was designated as pMD19T-Cap. To calculate the plasmid DNA copy number, the following formula was utilized:


$$\text{Plasmid copy number }\left(\text{copies}/\text{μL}\right)=\frac{\text{Plasmid concentration }\left(\text{ng}/\text{μL}\right)\;\times \;6.02\times \;10^{23}\times \;10^{-9}}{660\;\left(\text{g}/\text{mol}\right)\;\times \;\left[\text{lasmid length }\left(\text{bp}\right)\;+\text{ fragment length }\left(\text{bp}\right)\right]}$$

### 2.4 Primer and probe design and synthesis

The genomic sequences of 35 GETV strains, published in the GenBank nucleotide sequence database on NCBI, were downloaded for alignment analysis using the Megalign program in DNAStar software. Following the manufacturer’s protocol of the DNA isothermal amplification kit (basic type) (Amplification Future Biotech Co. Ltd., Weifang, China), high amplification efficiency and detection sensitivity were achieved by selecting primers with lengths ranging from 30 to 35 bp, while optimizing the amplification product length between 150 and 500 bp. According to the instructions of the DNA isothermal amplification kit (colloidal gold type) (Amplification Future Biotech Co. Ltd., Weifang, China), a probe of 46–52 bp complemeary to the target fragment was designed between the optimal forward and reverse primers. The base at position 33 from the 5’ end of the probe was replaced with tetrahydrofuran (THF) as the recognition site of endonuclease IV (nfo). The 5’ end of the probe was labeled with the fluorescent group (FAM), and the 3’ end was modified with a blocking group (C3-spacer). After designing the probe, its specificity was verified through alignment on NCBI to avoid non-specific reactions. Furthermore, the 5’ end of the forward primer was labeled with biotin. In summary, we developed a set of molecular tools for RT-RAA, including one probe (nfo-probe), five forward primers (F1-F5), and five reverse primers (R1-R5) for screening. The primer pair used for RT-PCR (PCR-F/R) was based on the local standard “Technical Specification for GETV Isolation and Identification in Pigs” (DB51/T2906-2022) from Sichuan Province. qRT-PCR primers were designed using Primer Premier 5.0, targeting the most conserved region of the GETV Cap gene. These primers were screened using Oligo 7 software to select primers with minimal or no dimer formation. All primers and probes were synthesized by Sangon Biotech Co. Ltd. (Shanghai, China), and their detailed information is provided in **Table 1**.

**Table 1:** The primers and probes of the GETV-based RT-RAA-LFD, RT-PCR, and qRT-PCR assays.

Primers/Probes	Nucleotide sequences(5’→3’)	Primer/probe position^a^
F1	CCCTGACGACCAAGCAAAATGGTAAAGCAC	7696-7725
F2	GACGACCAAGCAAAATGGTAAAGCACCGAA	7700-7729
F3	ACGACCAAGCAAAATGGTAAAGCACCGAAG	7701-7730
F4	CAAAATGGTAAAGCACCGAAGAAGCCGAAG	7710-7739
F5	AACCACCACCTAAGCAGAAGAACCCGGCTA	7801-7830
R1	AGCTTGACCTCGAAGATGCAATCATTCTCT	7874-7903
R2	ATTGTGAACCTGCCACCGCTGTACTGCACT	8126-8155
R3	CTGGTTTACCTGCGCCTGTCGGGATTGTGA	8149-8178
R4	ATCATTCTCTATCTTCATGCACATGCGTTC	7854-7883
R5	ATTCTCTATCTTCATGCACATGCGTTCCCT	7851-7880
Final F	Biotin-CCCTGACGACCAAGCAAAATGGTAAAGCAC	7696-7725
Final R	ATTGTGAACCTGCCACCGCTGTACTGCACT	8126-8155
nfo-probe	FAM-ATGTGCACTGGTATCTGGGCGCACTCCAGGTC [THF]TACTTGCTCGATTTCT[C3-spacer]	8011-8059
PCR-F	ACCGAAGAAGCCGAAGAA	7724-7741
PCR-R	GCACTCRAGGTCATACTTG	8021-8039
qRT-PCR-F	CCTGCCTAGTCGGGGATAA	7927-7945
qRT-PCR-R	AATTGTAGTGCCCTTCTGGT	8093-8112

a The positions of probes and primers are referenced to the genomic sequence of GETV NM2022 strain (GenBank accession no. PP537546). FAM, 6-carboxyfluorescein; THF, tetrahydrofuran.

### 2.5 Agarose gel electrophoresis-based RT-RAA assay

The pMD19T-Cap recombinant plasmid was used as the template, with ddH_2_O serving as the negative control. A 50 μL reaction mixture was prepared according to the instructions of the DNA isothermal amplification kit (basic type) (Amplification Future Biotech Co. Ltd., Weifang, China). The following components were sequentially added to the dry powder: 29.4 μL of A buffer, 2 μL of forward primer (10 μmol/L), 2 μL of reverse primer (10 μmol/L), 5 μL of DNA template, 9.1 μL of ddH_2_O, and 2.5 μL of B buffer. After adding B buffer, the reaction tube was inverted 8–10 times to mix thoroughly, followed by centrifugation. The mixture was then placed in a T100 PCR thermal cycler (Bio-Rad, Hercules, CA, USA) at 37°C for 15 min. After the reaction, 50 μL of DNA extraction buffer (Tris-saturated phenol: chloroform: isopentanol = 25:24:1) was added to the amplification product. The solution was mixed and centrifuged at 12,000 rpm for 5 min. Finally, 5 μL of the supernatant was mixed with 2 μL of 6 × Loading buffer and analyzed by 2% agarose gel electrophoresis for identification.

### 2.6 RT-RAA-LFD assay

A 50 μL reaction mixture was prepared according to the instructions of the DNA isothermal amplification kit (colloidal gold type) (Amplification Future Biotech Co. Ltd., Weifang, China). The following components were sequentially added to the dry powder: 29.4 μL of A buffer, 2 μL of forward primer (10 μmol/L), 2 μL of reverse primer (10 μmol/L), 4 μL of DNA template, 0.6 μL of nfo-probe (10 μmol/L), 9.5 μL of ddH_2_O, and 2.5 μL of B buffer. After adding B buffer, the reaction tube was inverted 8–10 times to mix thoroughly, followed by centrifugation. The mixture was then placed in a T100 PCR thermal cycler (Bio-Rad, Hercules, CA, USA) at 40°C for 15 min. After the reaction, the amplification product was 10-fold diluted with ddH_2_O. The diluted product was mixed thoroughly, and 60 μL of it was added to the sample wells of the LFD for visualization. The results were interpreted within 5 min by observing the control (C) and test (T) lines on the LFD. Interpretation of results: If red bands appear on both the C and T lines, the result is positive, indicating the presence of GETV nucleic acid in the sample. If a red band appears only on the C line, the result is negative, suggesting that the concentration of GETV nucleic acid in the sample is below the detection limit or absent. If neither the C line nor the T line shows a red band, the result is invalid.

### 2.7 RT-PCR and qRT-PCR assays

For the RT-PCR assay, a 20 μL reaction mixture was prepared according to the instructions of Taq MasterMix (Cwbio, Beijing, China), including 5 μL of 2 × Taq MasterMix, 0.5 μL of forward primer (10 μmol/L), 0.5 μL of reverse primer (10 μmol/L), 1 μL of DNA template, and 3 μL of ddH_2_O. Amplification was performed using a T100 Thermal Cycler (Bio-Rad, Hercules, CA, USA) with the following thermal cycling program: 95°C for 5 min; 95°C for 30 s; 40 cycles of 56°C for 30 s and 72°C for 30 s; and 72°C for 7 min.

For the qRT-PCR assay, a 25 μL reaction mixture was prepared according to the instructions of UltraSYBR Mixture (Cwbio, Beijing, China), including 12.5 μL of 2 × Ultra SYBR Mixture, 0.5 μL of forward primer (10 μmol/L), 0.5 μL of reverse primer (10 μmol/L), 2 μL of DNA template, and 9.5 μL of ddH_2_O. Amplification was carried out using the QIAquant 96 real-time PCR Thermal Cycler (Qiagen, Hilden, Germany) with the following thermal cycling program: 95°C for 10 min; and 40 cycles of 95°C for 15 s and 60°C for 1 min.

### 2.8 Optimization of the RT-RAA-LFD assay reaction conditions

RT-RAA amplification was performed with the optimal primer pair and probe, using 5.53 × 10^4^ copies/μL pMD19T-Cap recombinant plasmid as a template. To optimize the reaction temperature, the reaction was conducted at temperatures of 15°C, 20°C, 25°C, 30°C, 37°C, 40°C, 42°C, 45°C, and 50°C, with a constant reaction time of 15 min. After identifying the optimal reaction temperature, different reaction times of 1 min, 5 min, 10 min, 15 min, 20 min, 25 min, and 30 min were tested. The optimal reaction temperature and time were determined based on the intensity of the T-line color.

### 2.9 Specificity analysis

The specificity of the RT-RAA-LFD assay was assessed using nucleic acids from GETV, PEDV, PDCoV, inactivated CSFV, SVA, PCV2, and PRV as templates. A negative control, using ddH_2_O as the nucleic acid template, was also included.

### 2.10 Sensitivity analysis

The pMD19T-Cap recombinant plasmid was serially diluted 10-fold, achieving plasmid concentrations ranging from 5.53 × 10^9^ to 5.53 × 10^0^ copies/μL. Each dilution was used as a template, with ddH_2_O serving as the negative control. Detection was performed using RT-PCR, RT-RAA, and qRT-PCR assays under the optimal reaction conditions. The sensitivity of these assays was then compared.

### 2.11 Evaluation of diagnostic performance

A total of 30 serum samples, comprising both infected and control groups, were collected and mixed. From these, 21 samples were randomly selected for testing. Additionally, 60 tissue samples, including liver, kidney, spleen, and brain tissues from both infection and control groups, were pooled, and 45 samples were randomly selected for analysis. These included 15 liver samples, 10 spleen samples, 8 kidney samples, and 12 brain tissue samples. The selected serum and tissue samples were analyzed using RT-PCR, RT-RAA-LFD, and qRT-PCR assays. The results were interpreted blindly and then compared to evaluate the diagnostic performance of each assay.

### 2.12 Statistical analysis

The experimental data were processed and analyzed using IBM SPSS Statistics 27.0 software. Kappa statistics were employed to compare the diagnostic performance of experimentally infected samples, with statistical significance set at P< 0.05.

## 3 Results

### 3.1 Screening of the optimal primer pair for the RT-RAA-LFD assay

**Figure 2:**
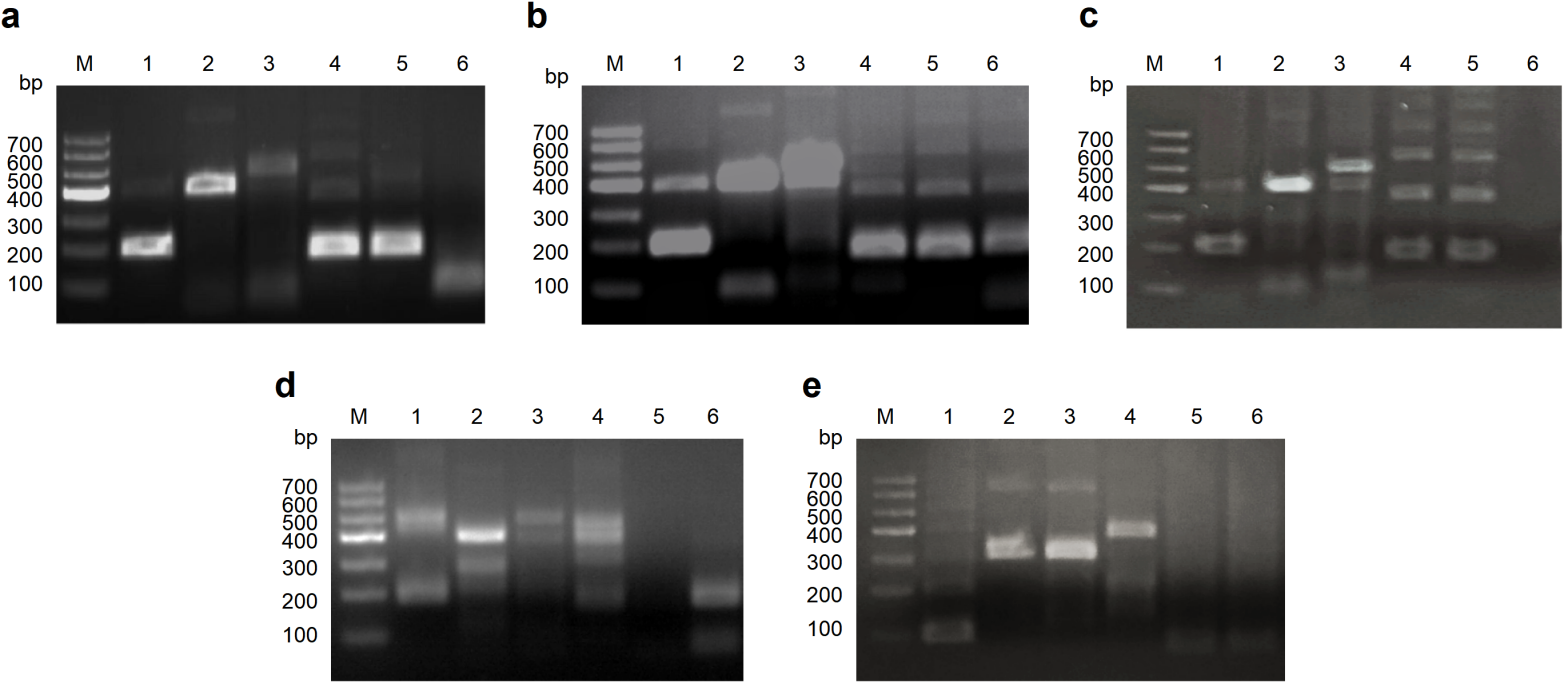
Screening of the optimal primer pair for the RT-RAA assays. **(a)** Screening of all reverse primers (R1-R5) using F1 as the forward primer. **(b)** Screening of all reverse primers (R1-R5) using F2 as the forward primer. **(c)** Screening of all reverse primers (R1-R5) using F3 as the forward primer. **(d)** Screening of all reverse primers (R1-R5) using F4 as the forward primer. **(e)** Screening of all reverse primers (R1-R5) using F5 as the forward primer.

Through genomic sequence alignment, we designed five forward primers (F1-F5) and five reverse primers (R1-R5) within conserved regions of the Cap gene. The primers were evaluated using the agarose gel electrophoresis-based RT-RAA assay, and the optimal forward and reverse primer pair F1/R2 was selected (**Figure 2**). The positions of the final primer pair and nfo-probe in the Cap gene are illustrated in **Supplementary Figure S1**.

### 3.2 Optimization of the RT-RAA-LFD assay reaction conditions

**Figure 3:**
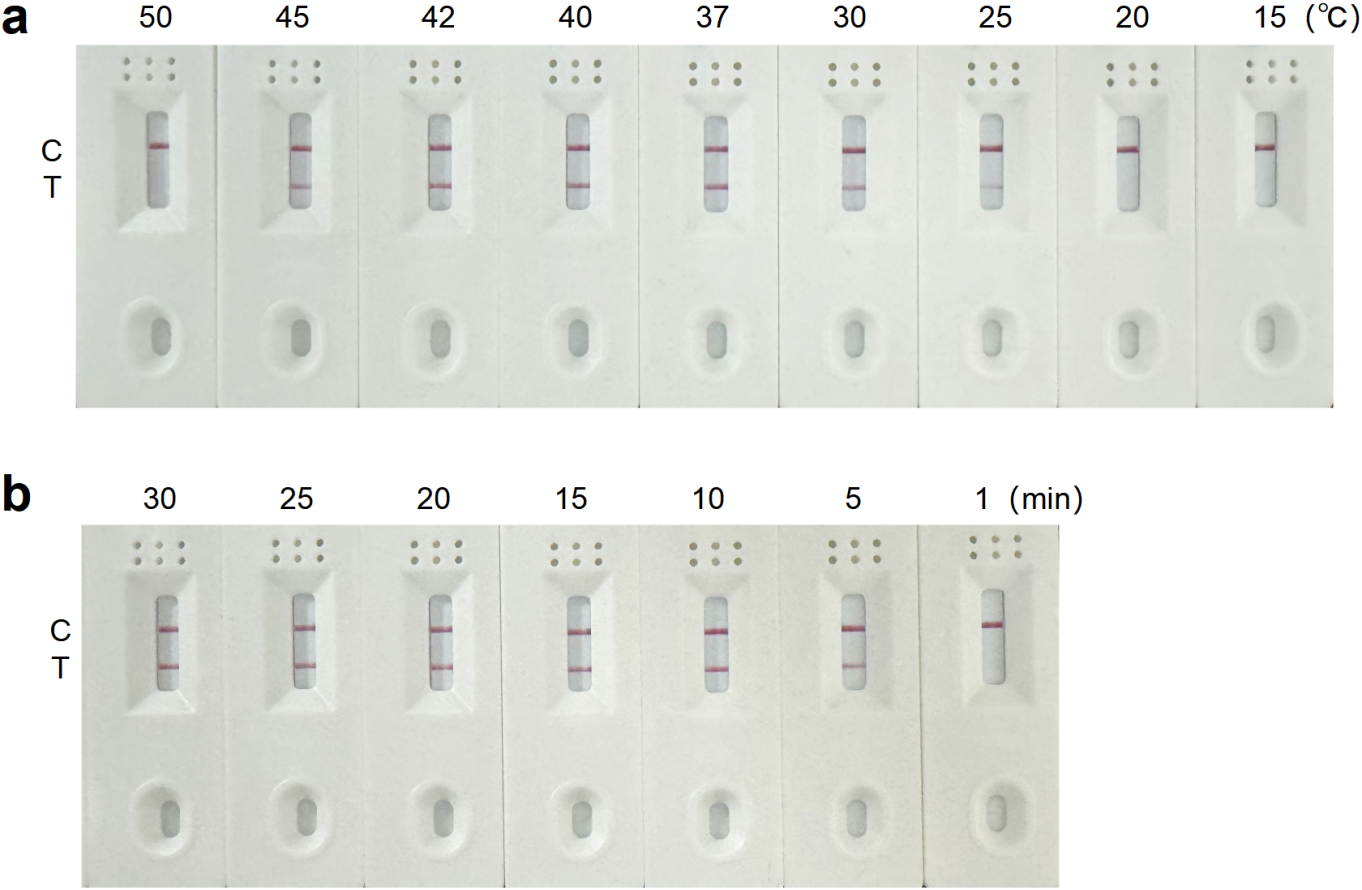
Optimization of the RT-RAA-LFD assay reaction conditions for GETV detection. **(a)** Reaction temperature optimization of the RT-RAA-LFD assay. **(b)** Reaction time optimization of the RT-RAA-LFD assay.

Using the pMD19T-Cap recombinant plasmid as the template, RT-RAA amplification was performed with the optimal forward primer, reverse primer and nfo-probe. The reactions were carried out at different temperatures, with a constant reaction time of 15 min. No band was observed on the T line below 25°C. The T line signal intensified from 25°C, reached maximum intensity at 40°C, and disappeared at 50°C, indicating 40°C as the optimal temperature (**Figure 3a**). Reactions at the optimal temperature of 40°C were conducted for different durations. Bands appeared on the T line at 5 min, deepened at 10 min, and did not intensify with longer reaction times. Sensitivity analysis showed that the LOD achieved at 40°C for 10 min was higher than that obtained at 40°C for 15 min (**Supplementary Figure S2**). Therefore, a reaction time of 15 min was selected to ensure complete amplification (**Figure 3b**). In conclusion, the optimal reaction conditions for the RT-RAA-LFD assay were 40°C for 15 min.

### 3.3 Specificity analysis of the RT-RAA-LFD assay

**Figure 4:**
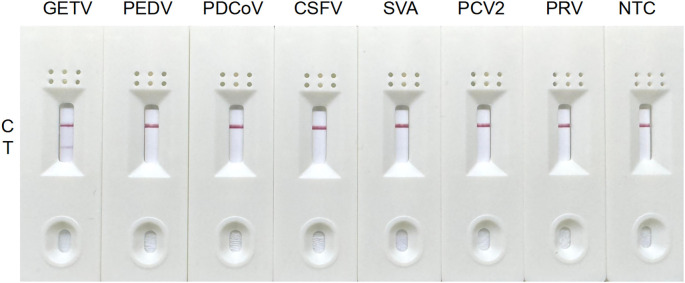
Specificity analysis of the RT-RAA-LFD assay for GETV detection.

The RT-RAA-LFD assay was assessed in triplicate using nucleic acids from clinically relevant swine pathogens, including GETV, PEDV, PDCoV, inactivated CSFV, SVA, PCV2, and PRV. A positive signal, indicated by red bands on both C line and T line, was observed exclusively for GETV. Other viruses and the negative control yielded negative results, confirming the high specificity of the RT-RAA-LFD assay for GETV detection (**Figure 4**).

### 3.4 Sensitivity analysis of the RT-RAA-LFD assay

**Figure 5:**
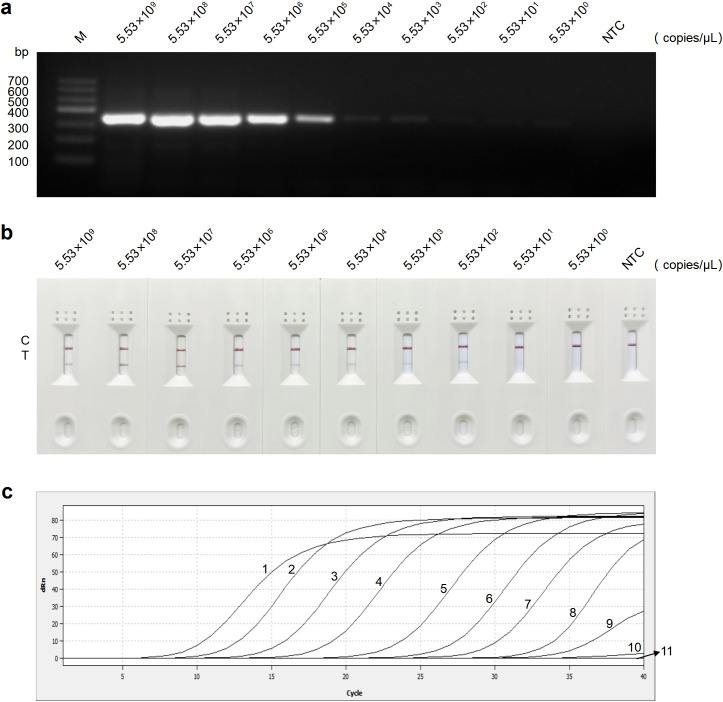
Sensitivity analysis of the RT-PCR, RT-RAA-LFD, and qRT-PCR assays for GETV detection. **(a)** Sensitivity analysis of the RT-PCR assay. **(b)** Sensitivity analysis of the RT-RAA-LFD assay. **(c)** Sensitivity analysis of the qRT-PCR assay. Curves 1-11: 5.53 × 10^9^ to 5.53 × 10^0^ copies/μL of plasmid concentration and negative control, respectively. NTC, no template control.

Detection using RT-PCR, RT-RAA-LFD, and qRT-PCR assays was performed in triplicate on recombinant plasmid with concentrations ranging from 5.53 × 10^9^ to 5.53 × 10^0^ copies/μL. The results revealed a gradual decrease in amplification signal as the plasmid copy number decreased. The detection ranges for plasmid concentrations by RT-PCR, RT-RAA-LFD, and qRT-PCR assays were 5.53 × 10^9^ to 5.53 × 10^3^ copies/μL (**Figure 5a**), 5.53 × 10^9^ to 5.53 × 10^2^ copies/μL (**Figure 5b**), and 5.53 × 10^9^ to 5.53 × 10^1^ copies/μL (**Figure 5c**), respectively. In conclusion, the RT-RAA-LFD assay established in this study demonstrated excellent sensitivity and a broad detection range.

### 3.5 Analysis of experimentally infected samples using RT-RAA-LFD assay

Blood samples from mice were tested using RT-PCR, RT-RAA-LFD, and qRT-PCR assays. The RT-RAA-LFD assay established in this study demonstrated that, out of 21 blood samples, 10 were positive and 11 were negative, with results fully consistent with those of the RT-PCR and qRT-PCR assays, as shown in **Table 2**. Furthermore, tissue samples from mice were tested using the same assays. As presented in **Table 3**, the Kappa value for consistency between RT-RAA-LFD and RT-PCR was 0.776 (P< 0.001), with a concordance rate of 95.6% (43/45). The Kappa value for consistency between RT-RAA-LFD and qRT-PCR was 0.845 (P< 0.001), with a concordance rate of 97.8% (44/45). These findings indicated that the RT-RAA-LFD assay exhibited strong clinical diagnostic performance and was suitable for the detection of GETV.

**Table 2:** Comparison of the clinical diagnostic performance between the RT-PCR, RT-RAA-LFD, and qRT-PCR assays for GETV detection on blood samples.

Clinical sample	RT-PCR		RT-RAA-LFD		qRT-PCR		Coincidence rate
	Positive	Negative	Positive	Negative	Positive	Negative	
Blood	10	11	10	11	10	11	100%

**Table 3:** Comparison of the clinical diagnostic performance between the RT-PCR, RT-RAA-LFD, and qRT-PCR assays for GETV detection on tissue samples.

Assay		RT-RAA-LFD			Kappa	P-value	Coincidence rate
		Positive	Negative	Total			
RT-PCR	Positive	39	0	39	0.776	<0.001	95.6%
	Negative	2	4	6			
	Total	41	4	45			
qRT-PCR	Positive	41	1	42	0.845	<0.001	97.8%
	Negative	0	3	3			
	Total	41	4	45			

## 4 Discussion

Since the first isolation of GETV from Aedes albopictus mosquitoes in Malaysia in 1955, the virus has rapidly spread worldwide, with a broad geographical distribution (Jian et al., 2024). It has since been reported in numerous countries, with the majority of cases concentrated in pigs and horses. Currently, there is no approved commercial vaccine for the prevention and control of GETV. Therefore, there is an urgent need to develop rapid and effective diagnostic methods for GETV, which will provide a foundation for its detection and the prevention and control of outbreaks.

Recent advancements in isothermal amplification technologies have led to their widespread application in the development of diagnostic methods for pathogens in animals, plants, bacteria, and parasites (Mao et al., 2023). RAA, a novel isothermal amplification technique, is an enhanced version of RPA. It can be performed under constant temperature conditions within 15–30 minutes, offering advantages such as a short reaction time, low temperature, and a simple protocol (Zhang et al., 2024). Lateral flow dipstick (LFD) is characterized by high sensitivity, portability, and ease of visualization, making them ideal for on-site diagnostic tools (Hao et al., 2022). The combination of RAA and LFD overcomes the limitations of conventional PCR methods, such as long reaction times, complex operation, and high instrument requirements. GETV is a single−stranded, positive−sense RNA virus with a genome (~12 kb) comprising 5’ and 3’ untranslated regions, two open reading frames (ORF1 and ORF2), and a poly(A) tail (Ren et al., 2020). ORF1, located at the 5’ end, encodes four non-structural proteins (NSP1-NSP4), whereas ORF2, positioned at the 3’ end, encodes five structural proteins (Cap, E1, E2, E3, and 6K) (Lan et al., 2025). The Cap protein forms the viral nucleocapsid and is crucial for genomic RNA encapsidation, viral budding, and virion assembly (Aggarwal et al., 2017). Due to its high sequence conservation and structural stability, the Cap protein is an attractive molecular target for GETV detection and basic virological research (Jian et al., 2024; Lan et al., 2025). In this study, we established a RT-RAA-LFD assay targeting GETV Cap gene, and could be completed at 40°C for 15 min. The developed RT-RAA-LFD assay was used to detect other clinically relevant swine pathogens, showing no non-specific amplification of other viruses, indicating that the assay has excellent specificity. Furthermore, sensitivity analysis using recombinant plasmid with concentrations ranging from 5.53 × 10^9^ to 5.53 × 10^0^ copies/μL revealed that the sensitivity of RT-RAA-LFD was lower than that of RT-PCR (5.53 × 10^3^ copies/μL) and slightly higher than that of qRT-PCR (5.53 × 10^1^ copies/μL). Its performance was comparable to the previously reported one-tube RT-LAMP-PfAgo assay (1 × 10^2^ copies/μL) used for detecting the GETV NSP1 gene (Liu et al., 2025). Additionally, the RT-RAA-LFD assay was validated for its high sensitivity in detecting GETV RNA in mouse samples. Although one specimen with a viral load between 5.53 × 10^1^ and 5.53 × 10^2^ copies/μL was positive by qRT−PCR but negative by RT−RAA−LFD, the RT−RAA−LFD assay still shortened turnaround time compared to both RT−PCR and qRT−PCR. These results demonstrated that our RT−RAA−LFD assay exhibited good sensitivity and a broad detection range, providing a new approach for the rapid screening of GETV.

Although isothermal amplification technologies are widely used for pathogen detection, they have certain limitations. In the development of the RT-RAA-LFD assay, primer design and selection directly influence amplification efficiency, which is critical to the success of the entire experiment. Therefore, the appropriate primer combination is essential for achieving successful outcome (Li and Brownley, 2010). RAA primers typically require a length of 30–35 bp, which is considerably longer than conventional PCR primers. Unfortunately, there is no specialized software for designing primers that meet the RAA requirements, and even after multiple rounds of primer screening, errors within a certain range may still occur (Li et al., 2019). To ensure high amplification efficiency, we designed five sets of primers for screening to avoid primer-probe dimers, and selected the best primer-probe pair to establish the RT-RAA-LFD assay. However, the established assay still requires improvements. Specifically, the assay relied on TRIzol reagent for RNA extraction, which is inconvenient for field detection. The use of commercial one-step nucleic acid extraction kits or the development of a rapid nucleic acid extraction method could effectively simplify the detection process without compromising detection efficiency, making the RT-RAA-LFD assay more efficient and user-friendly (**Supplementary Table S1**) (Azmi et al., 2021; Zhang et al., 2021; Cui et al., 2023). Furthermore, the existing protocol remained a two-step workflow, requiring cDNA synthesis through reverse transcription. Developing a one-step RT-RAA-LFD format would further shorten turnaround time and simplify operation. Last, the diagnostic performance evaluation in our study utilized experimentally infected mouse samples, which primarily assessed analytical sensitivity. However, clinically derived porcine samples would better reflect actual field conditions, particularly given that all control pathogens included in this study are of porcine origin. Therefore, further validation studies employing porcine specimens are necessary to enhance both the experimental relevance and ecological validity of our findings.

In conclusion, we have developed a rapid and reliable RT-RAA-LFD assay targeting the GETV Cap gene, which demonstrated excellent performance in detecting GETV across various samples, including blood, liver, kidney, spleen, and brain. This assay holds great potential for GETV screening in the livestock industry, particularly in laboratories with limited resources and equipment, thereby contributing to public health safety. In addition, optimizing RNA extraction procedures and addressing cost-related challenges will enhance the efficiency and accessibility of RT-RAA-LFD assay, further solidifying its role in veterinary clinical diagnostics for GETV. The availability of a rapid RT-RAA-LFD assay combined with a simplified nucleic acid extraction procedure will strengthen GETV surveillance networks in China and internationally. This advancement will facilitate the early identification of GETV cases, prompt implementation of animal movement restrictions, and targeted vector-management strategies in high-risk locations, such as farms, slaughterhouses, and import-export ports, ultimately reducing the economic impact of potential future outbreaks.

## Data Availability

The raw data supporting the conclusions of this article will be made available by the authors, without undue reservation.

## References

[B1] Aggarwal M. Kaur R. Saha A. Mudgal R. Yadav R. Dash P. K.. (2017). Evaluation of antiviral activity of piperazine against Chikungunya virus targeting hydrophobic pocket of alphavirus capsid protein. Antiviral Res. 146, 102–111. doi: 10.1016/j.antiviral.2017.08.015, PMID: 28842264

[B2] Azmi I. Faizan M. I. Kumar R. Yadav S. R. Chaudhary N. Singh D. K.. (2021). A saliva-based RNA extraction-free workflow integrated with cas13a for SARS-coV-2 detection. Front. Cell Infect. Microbiol. 11. doi: 10.3389/fcimb.2021.632646, PMID: 33796478 PMC8009180

[B3] Cao X. Y. Qiu X. S. Shi N. Ha Z. Zhang H. Xie Y. B.. (2022). Establishment of a reverse transcription real-time quantitative PCR method for Getah virus detection and its application for epidemiological investigation in Shandong, China. Front. Microbiol. 13. doi: 10.3389/fmicb.2022.1009610, PMID: 36212868 PMC9538719

[B4] Cui H. Guan J. Y. Lu H. J. Liu J. Tu F. Zhang C.. (2023). Rapid onsite visual detection of orf virus using a recombinase-aided amplification assay. Life 13, 494. doi: 10.3390/life13020494, PMID: 36836851 PMC9968157

[B5] Fan X. X. Li L. Zhao Y. G. Liu Y. T. Liu C. J. Wang Q. H.. (2020). Clinical validation of two recombinase-based isothermal amplification assays (RPA/RAA) for the rapid detection of African swine fever virus. Front. Microbiol. 11. doi: 10.3389/fmicb.2020.01696, PMID: 32793160 PMC7385304

[B6] Fukunaga Y. Kumanomido T. Imagawa H. Ando Y. Kamada M. Wada R.. (1981). Isolation of picornavirus from horses associated with Getah virus infection. Nihon Juigaku Zasshi. 43, 569–572. doi: 10.1292/jvms1939.43.569, PMID: 6279949

[B7] Hao L. W. Yang W. T. Xu Y. Cui T. M. Zhu G. Q. Zeng W. W.. (2022). Engineering light-initiated afterglow lateral flow immunoassay for infectious disease diagnostics. Biosens. Bioelectron. 212, 114411. doi: 10.1016/j.bios.2022.114411, PMID: 35623251 PMC9119864

[B8] Hubálek Z. Rudolf I. Nowotny N. (2014). Arboviruses pathogenic for domestic and wild animals. Adv. Virus Res. 89, 201–275. doi: 10.1016/B978-0-12-800172-1.00005-7, PMID: 24751197

[B9] Jian Z. J. Jiang C. Y. Zhu L. Li F. Q. Deng L. S. Ai Y. R.. (2024). Infectivity and pathogenesis characterization of getah virus (GETV) strain via different inoculation routes in mice. Heliyon 10, e33432. doi: 10.1016/j.heliyon.2024.e33432, PMID: 39040396 PMC11260979

[B10] Karabatsos N. (1978). Supplement to International Catalogue of Arboviruses including certain other viruses of vertebrates. Am. J. Trop. Med. Hyg. 27, 372–440. doi: 10.4269/ajtmh.1978.27.372, PMID: 646031

[B11] Lan J. H. Duan L. L. Liu X. Y. Zhou Y. Zeng B. T. Chen S. Y.. (2025). Seroprevalence of Getah virus in pigs in Southeast China determined with a recombinant Cap protein-based indirect ELISA. Front. Microbiol. 16. doi: 10.3389/fmicb.2025.1547670, PMID: 40034493 PMC11872902

[B12] Lan J. H. Fang M. T. Duan L. L. Liu Z. Wang G. G. Wu Q.. (2024). Novel porcine getah virus from diarrheal piglets in Jiangxi province, China: prevalence, genome sequence, and pathogenicity. Animals 14, 2980. doi: 10.3390/ani14202980, PMID: 39457910 PMC11503733

[B13] Li K. Brownley A. (2010). Primer design for RT-PCR. Methods Mol. Biol. 630, 271–299. doi: 10.1007/978-1-60761-629-0_18, PMID: 20301004

[B14] Li J. Macdonald J. von Stetten F. (2019). Review: a comprehensive summary of a decade development of the recombinase polymerase amplification. Analyst 144, 31–67. doi: 10.1039/c8an01621f, PMID: 30426974

[B15] Li X. D. Qiu F. X. Yang H. (1992). Isolation of Getah virus from mosquitos collected on Hainan Island, China, and results of a serosurvey. Southeast Asian J. Trop. Med. Public Health 23, 730–734., PMID: 1338481

[B16] Li B. Wang H. Y. Liang G. D. (2022). Getah virus (Alphavirus): an emerging, spreading zoonotic virus. Pathogens 11, 945. doi: 10.3390/pathogens11080945, PMID: 36015065 PMC9416625

[B17] Lin H. Zhao S. Liu Y. H. Shao L. Ye Y. Y. Jiang N. Z.. (2022). Rapid visual detection of plasmodium using recombinase-aided amplification with lateral flow dipstick assay. Front. Cell Infect. Microbiol. 12. doi: 10.3389/fcimb.2022.922146, PMID: 35811679 PMC9263184

[B18] Liu H. Hu J. Li L. X. Lu Z. S. Sun X. T. Lu H. J.. (2023). Seroepidemiological investigation of Getah virus in the China-Myanmar border area from 2022-2023. Front. Microbiol. 14. doi: 10.3389/fmicb.2023.1309650, PMID: 38163077 PMC10755881

[B19] Liu Z. Yang F. S. Fang M. T. Wu Q. Fan K. Huang D. Y.. (2025). Rapid and sensitive one-tube detection of getah virus using RT-LAMP combined with pyrococcus furiosus argonaute. Vet. Sci. 12, 93. doi: 10.3390/vetsci12020093, PMID: 40005853 PMC11860293

[B20] Liu H. Zhang X. Li L. X. Shi N. Sun X. T. Liu Q.. (2019). First isolation and characterization of Getah virus from cattle in northeastern China. BMC Vet. Res. 15, 320. doi: 10.1186/s12917-019-2061-z, PMID: 31488162 PMC6729113

[B21] Lu G. Chen R. A. Shao R. Dong N. Liu W. Q. Li S. J. (2020). Getah virus: An increasing threat in China. J. Infect. 80, 356–358. doi: 10.1016/j.jinf.2019.11.016, PMID: 31790706

[B22] Lu G. Ou J. J. Ji J. Z. Ren Z. X. Hu X. Wang C. Y.. (2019). Emergence of getah virus infection in horse with fever in China 2018. Front. Microbiol. 10. doi: 10.3389/fmicb.2019.01416, PMID: 31281304 PMC6596439

[B23] Mao X. L. Xu M. H. Luo S. Y. Yang Y. Zhong J. Y. Zhou J. W.. (2023). Advancements in the synergy of isothermal amplification and CRISPR-cas technologies for pathogen detection. Front. Bioeng. Biotechnol. 11. doi: 10.3389/fbioe.2023.1273988, PMID: 37885449 PMC10598474

[B24] Mao L. J. Ying J. X. Selekon B. Gonofio E. Wang X. X. Nakoune E.. (2022). Development and characterization of recombinase-based isothermal amplification assays (RPA/RAA) for the rapid detection of monkeypox virus. Viruses 14, 2112. doi: 10.3390/v14102112, PMID: 36298667 PMC9611073

[B25] Morita K. Igarashi A. (1984). Oligonucleotide fingerprint analysis of strains of Getah virus isolated in Japan and Malaysia. J. Gen. Virol. 65, 1899–1908. doi: 10.1099/0022-1317-65-11-1899, PMID: 6094708

[B26] Nemoto M. Bannai H. Tsujimura K. Kobayashi M. Kikuchi T. Yamanaka T.. (2015). Getah virus infection among racehorses, Japan 2014. Emerg. Infect. Dis. 21, 883–885. doi: 10.3201/eid2105.141975, PMID: 25898181 PMC4412242

[B27] Ren T. W. Mo Q. R. Wang Y. X. Wang H. Nong Z. R. Wang J. L.. (2020). Emergence and phylogenetic analysis of a getah virus isolated in southern China. Front. Vet. Sci. 7. doi: 10.3389/fvets.2020.552517, PMID: 33344520 PMC7744783

[B28] Shi N. Zhu X. Y. Qiu X. S. Cao X. Y. Jiang Z. Y. Lu H. J.. (2022). Origin, genetic diversity, adaptive evolution and transmission dynamics of Getah virus. Transbound Emerg. Dis. 69, e1037–e1050. doi: 10.1111/tbed.14395, PMID: 34812572

[B29] Sun Y. W. Y. H. Zhang Y. S. Liu R. W. Li L. Y. Shi M. M. (2025). Outbreak and epidemic of Getah virus infection in swine by virulence-enhanced GIII variant in Henan, central China in 2024. Virulence 16, 2530661. doi: 10.1080/21505594.2025.2530661, PMID: 40653751 PMC12269664

[B30] Wu X. H. Yao Z. Q. Zhao Q. Q. Chen S. Hu Z. Z. Xie Z.. (2022). Development and application of a reverse-transcription recombinase-aided amplification assay for subgroup J Avian leukosis virus. Poult. Sci. 101, 101743. doi: 10.1016/j.psj.2022.101743, PMID: 35240352 PMC8889409

[B31] Yang T. Li R. Hu Y. Yang L. Zhao D. Du L.. (2018). An outbreak of Getah virus infection among pigs in China 2017. Transbound Emerg. Dis. 65, 632–637. doi: 10.1111/tbed.12867, PMID: 29575687

[B32] Zhang Y. H. Li Q. M. Guo J. Q. Li D. L. Wang L. Wang X.. (2021). An isothermal molecular point of care testing for African swine fever virus using recombinase-aided amplification and lateral flow assay without the need to extract nucleic acids in blood. Front. Cell Infect. Microbiol. 11. doi: 10.3389/fcimb.2021.633763, PMID: 33816338 PMC8010139

[B33] Zhang Z. S. Zhang Z. C. Wang C. G. Zhai X. H. Wang W. J. Chen X.. (2024). Detection method for reverse transcription recombinase-aided amplification of avian influenza virus subtypes H5, H7, and H9. BMC Vet. Res. 20, 203. doi: 10.1186/s12917-024-04040-9, PMID: 38755641 PMC11097555

[B34] Zhao J. Dellicour S. Yan Z. Q. Veit M. Gill M. S. He W. T.. (2023). Early genomic surveillance and phylogeographic analysis of getah virus, a reemerging arbovirus, in livestock in China. J. Virol. 97, e0109122. doi: 10.1128/jvi.01091-22, PMID: 36475767 PMC9888209

