# Supplementary material for: Establishment of reverse transcription recombinase-aided amplification with lateral flow dipstick for the rapid visual detection of Getah virus
Source: Front Cell Infect Microbiol. 2025 Aug 13;15:1631048. doi: 10.3389/fcimb.2025.1631048 (PMC12380932; doi:10.3389/fcimb.2025.1631048)
Supplement: Supplementary file 1 [file Supplementaryfile1.docx]

Figure S1. The positions of the final primer pair and nfo-probe in Cap gene of GETV. The GenBank accession numbers for various GETV strains are provided on the left side of the figure. The dots represent highly conserved nucleotide residues. The red and blue dashed boxes indicate the positions of the forward and reverse primers, respectively. The green dashed box shows the position of the nfo-probe.


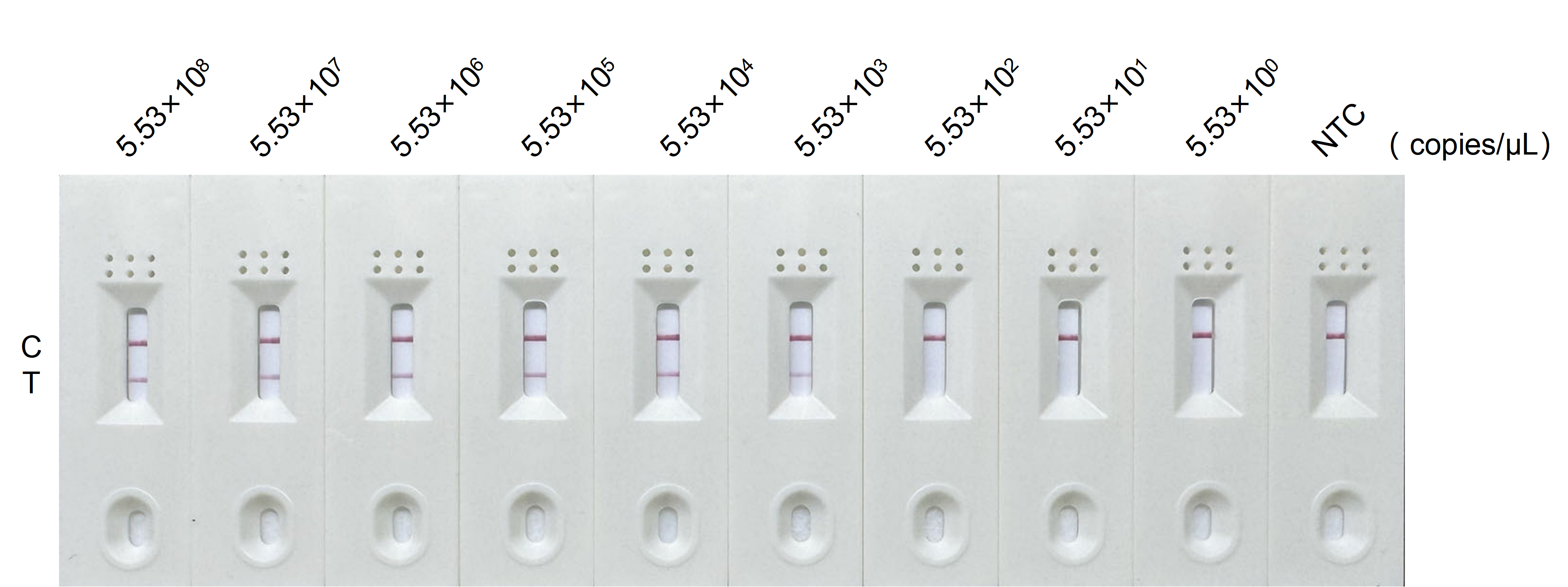


Figure S2. Sensitivity analysis of the RT-RAA-LFD at 40℃ for 10 min. NTC, no template control.

Table S1. Summary of simplified nucleic acid extraction in RPA/RAA viral detection

| Virus | Samples | simplified nucleic acid extraction method | Detection methods | Limit of Detection | References |
| --- | --- | --- | --- | --- | --- |
| SARS-CoV-2 | Saliva samples | • Proteinase K (1.25 mg/ml), NAC (0.5%), and Triton X-100 (0.5%)  • 37 ℃ for 10 min and 95 ℃ for 5 min | RPA | 200 copies/reaction | Azmi et al., 2021 |
| African swine fever virus | Blood samples | • Diluted with PBS in a ratio of 1:3  • 100 ℃ for 5 min | RAA | 10 copies/μL | Zhang et al., 2021 |
| Orf virus | Skin tissues samples | • cut into small pieces (0.2-0.3 g) using ophthalmic scissors  • placed directly in 100 µL MightyPrep reagent for DNA (TaKaRa, Dalian, China)  • 95 °C for 10 min | RAA | 13 copies/reaction | Cui et al., 2023 |

Azmi, I., Faizan, M.I., Kumar, R., Yadav, S.R., Chaudhary, N., Singh, D.K., et al. (2021). A Saliva-Based RNA Extraction-Free Workflow Integrated With Cas13a for SARS-CoV-2 Detection. *Frontiers in Cellular and Infection Microbiology* 11. doi: 10.3389/fcimb.2021.632646

Cui, H., Guan, J.Y., Lu, H.J., Liu, J., Tu, F., Zhang, C., et al. (2023). Rapid Onsite Visual Detection of Orf Virus Using a Recombinase-Aided Amplification Assay. Life-Basel 13. doi: 10.3390/life13020494

Zhang, Y.H., Li, Q.M., Guo, J.Q., Li, D.L., Wang, L., Wang, X., et al. (2021). An Isothermal Molecular Point of Care Testing for African Swine Fever Virus Using Recombinase-Aided Amplification and Lateral Flow Assay Without the Need to Extract Nucleic Acids in Blood. Frontiers in Cellular and Infection Microbiology 11. doi: 10.3389/fcimb.2021.633763
